# Supplementary material for: Micropropagation of Seed-Derived Clonal Lines of the Endangered Agave marmorata Roezl and Their Compatibility with Endophytes
Source: Biology (Basel). 2022 Sep 29;11(10):1423. doi: 10.3390/biology11101423 (PMC9598202; doi:10.3390/biology11101423)
Supplement: Supplementary file 1 [file biology-11-01423-s001.zip › biology-1868250-supplementary.pdf]

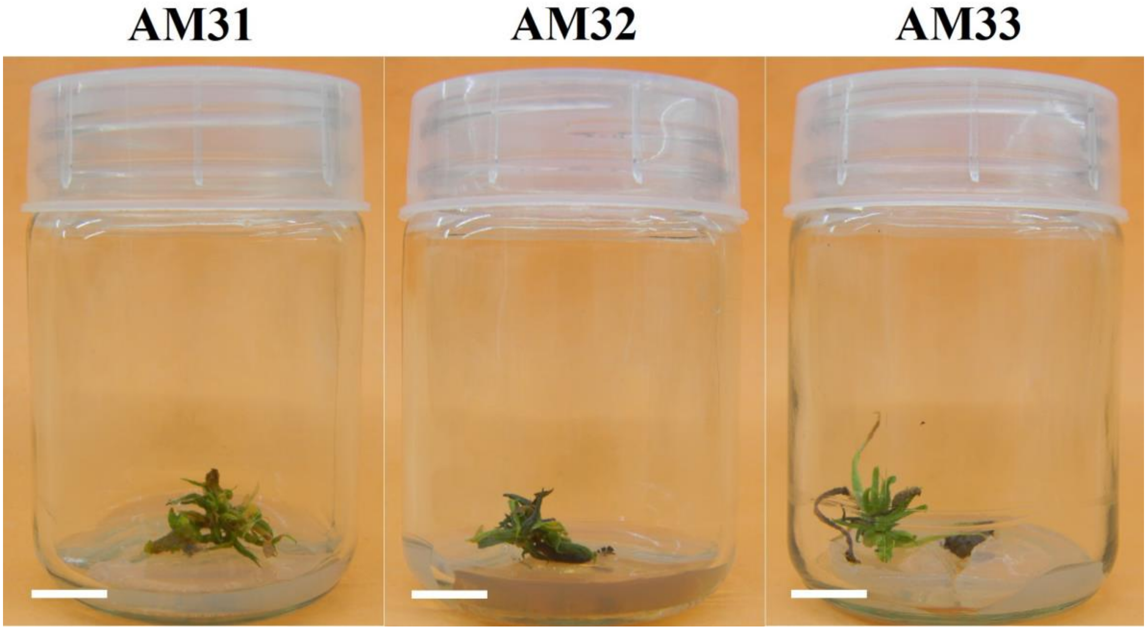

Supplementary Figure S1. Seedlings of the all-clonal lines in MS medium without BAP did not multiply shoots. Seedlings developed an attempt to form a mature plant a "mature plant" that did not grow in AM31, AM32, and AM33 selected lines. Bars= 1 cm.
